# Precisamos de Novos Parâmetros Ecocardiográficos para Transplantados sem Rejeição?

**DOI:** 10.36660/abc.20240452

**Published:** 2024-09-10

**Authors:** Alberto Rodolpho Hüning, Vitor Emer Egypto Rosa

**Affiliations:** 1 Universidade de São Paulo Faculdade de Medicina Hospital das Clínicas HCFMUSP São Paulo SP Brasil Instituto do Coração (InCor), Hospital das Clínicas HCFMUSP, Faculdade de Medicina, Universidade de São Paulo, São Paulo, SP - Brasil

**Keywords:** Transplante Cardíaco, Ecocardiograma, Strain Longitudinal

## Introdução

O transplante cardíaco representa a principal modalidade terapêutica para pacientes com insuficiência cardíaca terminal que não respondem ao manejo médico e cirúrgico maximizado.^1^ O Brasil possui um dos mais extensos sistemas de saúde pública para transplante de órgãos do mundo, com quase todos os procedimentos realizados no âmbito do Sistema Único de Saúde (SUS). Contudo, ainda faltam estudos que validem parâmetros ecocardiográficos normais nesta população, e este foi o tema do estudo realizado por Dall’Orto et al.^2^

### Ecocardiografia transtorácica em transplante cardíaco

A ecocardiografia transtorácica é essencial na avaliação de receptores de transplante cardíaco, embora com limitações inerentes. Embora a normalização da fração de ejeção do ventrículo esquerdo (FEVE) pós-transplante cardíaco esteja associada a um prognóstico favorável, existem limitações significativas na avaliação da função sistólica e diastólica do ventrículo esquerdo (VE) e do ventrículo direito, massa ventricular esquerda, doença cardíaca valvular, hipertensão arterial pulmonar e efusão pericárdica.^3 , 4^

Após o transplante, a biópsia endomiocárdica é realizada rotineiramente para vigilância da rejeição celular e humoral, a partir da segunda semana e aos 30 dias, 90 dias, 6 meses e 12 meses depois, ou quando há suspeita (p. ex., novo início de disfunção ventricular). Além disso, a biópsia endomiocárdica também é realizada para diagnosticar outras patologias, como miocardite, cardiomiopatias infiltrativas, insuficiência cardíaca de início recente, neoplasias cardíacas, arritmias ventriculares inexplicáveis, cardiomiopatia hipertrófica e continua sendo o padrão ouro para detectar rejeição aguda de aloenxerto.^5 , 6^

### Speckle Tracking e novas modalidades de ecocardiografia

As modalidades emergentes avançaram, mas a sua aplicação em receptores de transplante cardíaco é limitada. O strain longitudinal global (SLG) avaliado por meio do *speckle tracking* emergiu como um complemento valioso para a avaliação da função ventricular esquerda, oferecendo maior reprodutibilidade em comparação com a medição tradicional da FEVE.^7^ O strain longitudinal é utilizado para avaliar a função sistólica global do VE por meio da ecocardiografia, mostrando-se altamente útil para estratificação prognóstica em diversas doenças e para detecção precoce de envolvimento miocárdico. O SLG é a medida mais comumente usada, e o pico do SLG descreve as alterações relativas na deformação miocárdica do VE entre a diástole final e a sístole final. Um valor SLG normal é definido como maior ou igual a 20% em magnitude (ou ≤ -20% quando considerado negativo). Espera-se um valor em torno de -20% em um indivíduo normal, mas o limite inferior de normalidade pode variar de -11% a -18%, dependendo do software e equipamento utilizado. Em relação ao ventrículo direito (VD), o SLG é adaptado das medidas do VE. SLG VD normalmente se refere à média da parede livre e dos segmentos septais ou apenas da parede livre. Um SLG VD inferior a 20% (valor absoluto) é considerado anormal.^4^

Os índices de trabalho miocárdico (MWI) avaliam a demanda miocárdica de oxigênio e a função cardíaca, demonstrando superioridade sobre o SLG, pois é responsável pela deformação miocárdica juntamente com a pós-carga.^8 , 9^ É calculado incorporando a pressão ventricular esquerda não invasiva obtida por meio de manguito automático com o strain do VE, fornecendo índices associados à curva strain-pressão. O índice global de trabalho (GWI), o trabalho construtivo global (GCW), o desperdício global de trabalho (GWW) e a eficiência global do trabalho (GWE) são estimados a partir dos ciclos de pressão-deformação do LV. Os valores do trabalho miocárdico (MW) podem variar, variando de 1270 mmHg% (homens) e 1310 mmHg% (mulheres) para GWI a 238 mmHg (homens) e 239 mmHg (mulheres) para GWW.^10^

### Estudo atual

O estudo teve como objetivo principal avaliar o strain em pacientes transplantados cardíacos sem rejeição em comparação com indivíduos saudáveis. Uma redução significativa nos valores de strain foi observada no grupo transplantado (SLG VE11,99±2,74) em comparação ao grupo controle e aos padrões normais descritos nas diretrizes,^4^ apesar da FEVE preservada (64,52±6,88%). Além disso, o grupo transplantado apresentou diferenças em outras variáveis ecocardiográficas, como redução do strain da parede livre do VD e índices de trabalho miocárdico, maior tamanho do átrio esquerdo e aumento do índice de massa e espessura relativa da parede. Inicialmente foram incluídos 100 pacientes; no entanto, 35 foram excluídos devido à rejeição confirmada por biópsia.^2^

Os pontos fortes do estudo incluem uma metodologia robusta, comparando receptores de transplante sem rejeição (confirmada por biópsia endomiocárdica) a indivíduos saudáveis através de ecocardiografia realizada por três examinadores treinados, cegos para os resultados da biópsia. O uso de parâmetros ecocardiográficos inovadores do estudo NORRE^10^ é interessante, sendo o primeiro a aplicar esses parâmetros em transplantados sem rejeição (com mais de 50% dos casos por doença de Chagas) e compará-los com indivíduos saudáveis.

No entanto, o estudo tem limitações. As características basais da população saudável diferem substancialmente daquelas dos transplantados, principalmente no índice de massa corporal e na área de superfície corporal, comprometendo a avaliação de parâmetros não indexados. Além disso, faltam informações sobre indivíduos saudáveis, como comorbidades e uso de medicamentos. O tamanho da amostra é pequeno e um grupo controle maior poderia reduzir as diferenças nas características clínicas. Limitações menores incluíram a incapacidade de realizar medições da função diastólica em mais de 50% dos pacientes do grupo de transplante e a natureza unicêntrica do estudo.

As conclusões do estudo são intrigantes: a redução do SLG do VE e do VD e a diminuição do MWI em receptores de transplante sem rejeição apoiam a hipótese de potencial insuficiência cardíaca diastólica pós-operatória, com inflamação inicial como previamente evidenciado por Ingvarsson et al.^11^ No entanto, mais estudos com coortes maiores de pacientes, diversos softwares ecocardiográficos e desenho multicêntrico são necessários para validar esses achados.
