# Supplementary material for: Precisamos de Novos Parâmetros Ecocardiográficos para Transplantados sem Rejeição?
Source: Arq Bras Cardiol. 2024 Sep 10;121(8):e20240452. [Article in Portuguese] doi: 10.36660/abc.20240452 (PMC12092025; doi:10.36660/abc.20240452)
Supplement: Translation

# Translation: Do We Need New Echocardiographic Parameters for Transplant Recipients without Rejection?

**DOI:** 10.36660/abc.20240452i

**Authors:** Alberto Rodolpho Hüning, Vitor Emer Egypto Rosa

**Affiliations:** 1 São Paulo SP Brazil Instituto do Coração (InCor), Hospital das Clínicas HCFMUSP, Faculdade de Medicina, Universidade de São Paulo, São Paulo, SP - Brazil

**Keywords:** Heart Transplant, Echocardiography, Longitudinal Strain

## Introduction

Heart transplant represents the principal therapeutic modality for patients with end-stage heart failure unresponsive to maximized medical and surgical management.^1^Brazil boasts one of the most extensive public health systems for organ transplantation globally, with nearly all procedures conducted under the Unified Health System (SUS). However, there is still a lack of studies validating normal echocardiographic parameters in this population, and this was the topic of the study conducted by Dall’Orto et al.^2^

### Transthoracic echocardiography in heart transplant

Transthoracic echocardiography (TTE) is essential for assessing heart transplant recipients, albeit with inherent limitations. While normalization of left ventricular ejection fraction (LVEF) post-heart transplant is associated with a favorable prognosis, significant limitations exist in evaluating left ventricular and right ventricular systolic and diastolic function, left ventricular mass, valvular heart disease, pulmonary arterial hypertension, and pericardial effusion.^3 , 4^

After transplantation, endomyocardial biopsy is routinely performed for surveillance of cellular and humoral rejection, starting from the second week and at 30 days, 90 days, 6 months, and 12 months thereafter, or when there is suspicion (e.g., new onset ventricular dysfunction). Additionally, endomyocardial biopsy is also conducted to diagnose other pathologies such as myocarditis, infiltrative cardiomyopathies, recent-onset heart failure, cardiac neoplasms, unexplained ventricular arrhythmias, hypertrophic cardiomyopathy and remains the gold standard for detecting acute allograft rejection.^5 , 6^

### Speckle Tracking and new echocardiography modalities

Emerging modalities have advanced, yet their application in cardiac transplant recipients is limited. Global longitudinal strain (GLS) assessed via speckle tracking has emerged as a valuable adjunct for left ventricular (LV) function evaluation, offering enhanced reproducibility compared to traditional LVEF measurement.^7^Longitudinal strain is used to evaluate the global systolic function of the LV via echocardiography, proving highly useful for prognostic stratification in various diseases and for detecting early myocardial involvement. GLS is the most commonly used measure, and the GLS peak describes the relative changes in LV myocardial deformation between end-diastole and end-systole. A normal GLS value is defined as greater than or equal to 20% in magnitude (or ≤ -20% when considered negative). A value of around -20% is expected in a normal individual, but the lower limit of normality can range from -11% to -18%, depending on the software and equipment used. Regarding the right ventricle (RV), GLS is adapted from LV measurements. RV GLS typically refers to the average of the free wall and septal segments or just the free wall. An RV GLS less than 20% (absolute value) is considered abnormal.^4^

Myocardial work indices (MWI) evaluate myocardial oxygen demand and cardiac function, demonstrating superiority over GLS as it accounts for myocardial deformation alongside afterload.^8 , 9^It is calculated by incorporating the non-invasive estimated left ventricular pressure obtained through an automatic cuff with the LV strain, providing indices associated with the strain-pressure curve. Global work index (GWI), global constructive work (GCW), global work waste (GWW), and global work efficiency (GWE) are estimated from LV pressure–strain loops. The values of myocardial work (MW) can vary, ranging from 1270 mmHg% (men) and 1310 mmHg% (women) for GWI to 238 mmHg (men) and 239 mmHg (women) for GWW.^10^

### Current Study

The study primarily aimed to evaluate strain in heart transplant patients without rejection compared to healthy individuals. A significant reduction in strain values was observed in the transplant group (LV GLS 11.99±2.74) compared to the control group and the normal standards described in guidelines,^4^despite preserved LVEF (64.52±6.88%). Additionally, the transplant group exhibited differences in other echocardiographic variables as reduced RV free wall strain and myocardial work indices, larger left atrial size, and increased mass index and relative wall thickness. Initially, 100 patients were included; however, 35 were excluded due to biopsy-confirmed rejection.^2^

The study's strengths include a robust methodology, comparing transplant recipients without rejection (confirmed by endomyocardial biopsy) to healthy individuals via echocardiography conducted by three trained examiners, with echocardiographers blinded to biopsy results. The use of innovative echocardiographic parameters from the NORRE study^10^is significant, being the first to apply these parameters in transplant recipients without rejection (with over 50% of cases due to Chagas disease) and comparing them with healthy individuals.

However, the study has limitations. The baseline characteristics of the healthy population differ substantially from those of the transplant recipients, particularly in body mass index and body surface area, jeopardizing the evaluation of non-indexed parameters. Additionally, there is a lack of information about healthy subjects, such as comorbidities and medication use. The sample size is small, and a larger control group could reduce differences in clinical characteristics. Minor limitations included the inability to perform diastolic function measurements in over 50% of the transplant group patients and the single-center nature of the study.

The study's conclusions are intriguing: Reduced GLS of the LV and RV and decreased MWI in transplant recipients without rejection support the hypothesis of potential diastolic heart failure post-surgery, with initial inflammation as previously evidenced by Ingvarsson et al.^11^However, further studies with larger patient cohorts, diverse echocardiographic software, and multicenter settings are necessary to validate these findings.
